# Induction, expression and characterisation of laccase genes from the marine-derived fungal strains *Nigrospora* sp. CBMAI 1328 and *Arthopyrenia* sp. CBMAI 1330

**DOI:** 10.1186/s13568-015-0106-7

**Published:** 2015-03-11

**Authors:** Michel Rodrigo Zambrano Passarini, Cristiane Angelica Ottoni, Cledir Santos, Nelson Lima, Lara Durães Sette

**Affiliations:** Divisão de Recursos Microbianos, CPQBA/UNICAMP, CP 6171, 13083-970 Campinas, SP Brazil; CEB-Centre of Biological Engineering, University of Minho, Campus of Gualtar, 4710-057 Braga, Portugal; Post-Graduate Programme in Agricultural Microbiology, Federal University of Lavras, Lavras, MG Brazil; Departamento de Bioquímica e Microbiologia, Instituto de Biociências - UNESP, Campus Rio Claro, Av. 24A, n°1515, 13506-900 Rio Claro, SP Brazil

**Keywords:** Copper sulphate, Gene expression, Laccase, Marine fungi

## Abstract

The capability of the fungi *Nigrospora* sp. CBMAI 1328 and *Arthopyrenia* sp. CBMAI 1330 isolated from marine sponge to synthesise laccases (Lcc) in the presence of the inducer copper (1–10 μM) was assessed. In a liquid culture medium supplemented with 5 μM of copper sulphate after 5 days of incubation, *Nigrospora* sp. presented the highest Lcc activity (25.2 U·L^−1^). The effect of copper on Lcc gene expression was evaluated by reverse transcriptase polymerase chain reaction. *Nigrospora* sp. showed the highest gene expression of Lcc under the same conditions of Lcc synthesis. The highest Lcc expression by the *Arthopyrenia* sp. was detected at 96 h of incubation in absence of copper. Molecular approaches allowed the detection of Lcc isozymes and suggest the presence of at least two undescribed putative genes. Additionally, Lcc sequences from the both fungal strains clustered with other Lcc sequences from other fungi that inhabit marine environments.

## Introduction

Marine environments host a huge diversity of microorganisms. Fungi constitute a large part of this microbiota and are also diverse and are important from ecological and biotechnological points of view (Panno et al. 2013). Sponge-derived fungi have repeatedly been shown as interesting sources of novel bioactive metabolites previously not found from terrestrial strains of the same species (Subramani et al. 2013), because they are adapted, amongst others factors, to the harsh marine environment (e.g., high pressure, low temperature, oligotrophic nutrients, high salinity) (Chen et al. 2011). Among the metabolites produced by marine fungi stand out enzymes, that represent enormous potential for the production of pharmaceutical compounds, food, beverages, aromas, fragrances, agrochemicals, and fine chemicals (Rocha et al. 2012).

Laccases (Lcc, EC 1.10.3.2) are found across kingdoms of life (e.g., plants, insects, bacteria and fungi) and, in the Nature act preferably on phenolic compounds. They belong to the class of oxidoreductases and to the family multicopper oxidases as well as ferroxidases, bilirubin oxidases and ascorbate oxidases (Ramos et al. 2011). Coding genes from this family appear to be redundant in fungal genomes, probably due to the different physiological roles played by their coding products and their regulation depending on environmental conditions (Ramos et al. 2011).

The widespread occurrence of Lcc in fungi and their versatility, especially in white-rot fungi, contribute to further research to obtain new sources of improved enzymes (Haibo et al. 2009). Although the copper catalytic centres are similar for all fungal Lcc, significant differences are observed on thermodynamic and kinetic properties depending on the microorganism (de Oliveira et al. 2009). The fungal Lcc often occur as multiple isoenzymes expressed under different growth conditions. Various strategies have been employed to improve the production of Lcc, such as medium optimization, isolation and breeding of high-producing strains, the utilization of inducers, and the heterologous expression of Lcc genes (Wang et al. 2013). Furthermore, most of Lcc are extracellular inducible enzymes, their rates of synthesis and activity being strongly dependent on the presence of suitable inductor which plays an important role in increasing their production (Kocyigit et al. 2012).

Reports investigating enzymes produced by marine-derived fungi, and their potential applications are very few. Recently, Chen et al. (2011) reported the production of laccases by a *Pestalotiopsis* strain. These authors obtained highest level of Lcc activity (2.0 U·l^−1^) under submerged growth using the untreated sugarcane bagasse. Bonugli-Santos et al. (2010) identified Lcc, manganese peroxidase (MnP) and lignin peroxidase (LiP) activities from marine-derived fungi such as *Aspergillus esclerotiorum*, *Cladosporium cladosporioides* and *Mucor racemosus*. Lcc are of particular interest with regard to potential industrial applications due to their ability to oxidize a wide range of toxic and polluting substrates, including polycyclic aromatic hydrocarbons (PAH) derived from petroleum, textile dyes and pesticides.

Fungal Lcc production is highly regulated by the media composition. Thus, medium optimization has become one of the main methods to enhance Lcc production. Carbon sources and copper ions are the two most critical factors in improving or stimulating Lcc (Wang et al. 2014). Different studies have shown that Lcc production are regulated by metal ions such as Cu^2+^ and Fe^3+^ by gene expression induction or through translational or post-translational regulation (Fonseca et al. 2010). Recently, Manavalan et al. (2013) described significantly increased (1.5 U ml^−1^) for Lcc production by *Ganoderma lucidum* when the culture medium was amended with 0.4 mM CuSO_4_. Nakade et al. (2013) using *Pycnoporus cinnabarinus* assessed the effect of different inducers (anicidine, catechol, guaiyacol, 2,5-xylidine, ferulic acid, ethanol ,H_2_O_2_, Cu^+2^, Mn^+2^, Fe^+2^) in different concentrations for Lcc activity. These authors concluded that the most effective inducer was CuSO_4_ (0.25 mM) where the Lcc activity detected (34.6 U·ml^−1^) was 20 times higher than in the absence of this inducer. In work by Lorenzo et al. (2006) the addition of copper to the growth medium stimulated Lcc production by *Trametes versicolor*. The cultures treated with 3.5 mM copper sulphate showed the highest Lcc activities of approximately 8 U·ml^−1^. This represented an increase of more than 12-fold in relation to the control culture and was nearly 25% higher than those obtained in the cultures with copper sulphate at 2 mM.

The fungal strains investigated in the present paper (*Nigrospora* sp. CBMAI 1328 and *Arthopyrenia* sp. CBMAI 1330) were isolated from the marine sponge *Dragmacidon reticulatum* and previously selected because of their capacity to synthesise great levels of Lcc (7.7 and 6.5 U·l^−1^), respectively (Passarini 2012). In the present work, the effect of copper sulphate on laccase synthesis by the strains was investigated to further enhance activity. In addition, the laccase gene diversity was determined.

## Materials and methods

### Microorganisms

The marine-derived fungi *Nigrospora* sp. and *Arthopyrenia* sp. were isolated from the sponge *Dragmacidon reticulatum* and identified according to Passarini et al. (2013). Both strains were deposited at the Brazilian culture collection “CBMAI/Coleção Brasileira de Micro-organismos de Ambiente e Indústria” (CPQBA/UNICAMP) under the accession number CBMAI 1328 and CBMAI 1330, respectively.

For the assays related to the present work the strains were maintained on Malt Extract Agar (MEA, Oxoid malt extract 20 g∙l^−1^, Sigma glucose monohydrate 20 g∙l^−1^, Oxoid bacto peptone1 g∙l^−1^, Oxoid agar-3 15 g∙l^−1^) at 4°C and subcultured every month.

### Culture conditions

#### Influence of copper sulphate on laccase gene expression

Lcc synthesis assays were performed in 250 ml Erlenmeyer flasks containing 100 ml of liquid culture medium (LCM: Oxoid malt extract 20 g l^−1^, Panreac NaCl 30 g l^−1^, Sigma copper sulphate 1–10 μM). Five plug disks of 8-mm diameter collected from the 7 days old colony of the fungi *Nigrospora* sp. CBMAI 1328 and *Arthopyrenia* sp. CBMAI 1330, previously growth on pre-adaption medium (PAM: LCM added by Oxoid agar-3 15 g∙l^−1^), were used as inocula. The submerged cultures were incubated in a Certomat rotary shaker (150 rpm) for 7 days at 28°C. Controls were carried out under identical conditions without copper sulphate.

### Molecular analyses

#### Genomic DNA isolation and amplification

Five 8-mm diameter plugs were cut as described above and used to inoculate 250 ml Erlenmeyer flasks with 100 ml GYP (Oxoid malt extract 3 g·l^−1^, Panreac glucose 10 g·l^−1^, Oxoid yeast extract 3 g·l^−1^ and Oxoid peptone 5 g·l^−1^). The cultures were incubated on a Certomat rotary shaker for 7 days at 28°C and 150 rpm. Mycelia from the *Nigrospora* sp. CBMAI 1328 and *Arthopyrenia* sp. CBMAI 1330 were frozen at −80°C and later used for genomic DNA extraction as previously described by Raeder and Broda (1985).

The degenerate primer pair LAC2FOR (5′-GGIACIWIITGGTAYCAYWSICA-3′) and LAC3REV (5′-CCRTGIWKRTGIAWIGGRTGIGG-3′) (Invitrogen) were used for amplifying two of the four copper binding regions (designated II and III) (Lyons et al. 2003). The housekeeping gene β-tubulin from the white rot fungus *Trametes versicolor* was used as reference (GenBank accession no. AY944858.1: 5′-CGGTGAGAGGCGTCGGACAC-3′). DNA amplification was performed using a thermocycler (Bio-Rad, MyCycler) with an initial denaturation of 3 min at 94°C; followed by 35 cycles of 0.5 min at 94°C, 0.5 min at x°C (x = Lcc: 48; β-tubulin = 47), 2 min at 72°C; final extension of 10 min at 72°C and cooled to 4°C. PCR reactions were done in a 50 μl final volume containing: 0.5 μl of DNA (97 ng μl^−1^), 1 μl of 200 mM deoxynucleotide triphosphates (Promega), 3 μl of MgCl_2_ solution (25 mM), 10 μl of GoTaq Flexi buffer (10×), 1 μl of each pair of primers (10 mM), 0.25 μl of enzyme GoTAQ Hot Start Polymerase (Promega) and MilliQ water.

Amplified products were visualized in 1.2% agarose gels stained with ethidium bromide. The bands of the expected sizes were removed and purified with GFX PCR DNA and Gel Band Purification Kit (GE Healthcare).

### Cloning, sequencing and sequence analysis

PCR products were cloned into pGEM-T Easy Vector (Promega) according to the manufacturer’s instructions and transformed into *E. coli* JM109 competent cells (Promega). About 15 clones per insert were sequenced with primers M13f (5′-CGCCAGGGTTTTCCCAGTCACGAC-3′) and M13r (5′-TTTCACACAGGAAACAGCTATGAC-3′). Amplified products were purified using GFX PCR DNA and Gel Band Purification Kit (GE Healthcare) for subsequent sequencing with DYEnamic ET Dye Terminator Cycler Sequencing Kit in an automated MegaBace DNA Analysis System 1000 (GE Healthcare) according to the manufacturer’s instructions.

The Phred/Phrap/CONSED software was used to assemble the sequences into a contig. Sequences were identified using BALSTn and BLASTx search (with Lcc gene references in the GenBank database) and aligned using ClustalX (Thompson et al. 1997). The determination of the intron was done according to Bonugli-Santos et al. (2010). For that end, the target sequences were aligned with known Lcc cDNA (e.g., *Neurospora crassa* AAA33591 or *Trametes versicolor* U44431) and were manually corrected with BIOEDIT 7 (Hall, 1999) and ClustalW (Thompson et al. 1997). The introns were discarded and the deduced protein sequences were determined before uploading the alignment into phylogenetic programmes. A distance approach using the Kimura 2-parameter model (Kimura 1980) as implemented in MEGA software version 5.0 (Tamura et al. 2011) was used as a substitution model.

Sequences were also used to create a picture of Lcc gene structures using FancyGene v1.4 (Rambaldi and Ciccarelli 2009).

### RNA extraction and RT-PCR

As described above five 8-mm diameter plugs of the *Nigrospora* sp. CBMAI 1328 and *Arthopyrenia* sp. CBMAI 1330 grown in PAM were inoculated in a 250 ml Erlenmeyer flask with 100 ml GYP and incubated on a Certomat rotary shaker for 5 days at 28°C and 150 rpm. For each fungus, biomass was then retrieved and carefully washed by vacuum filtration under sterile conditions for three times with 150 ml sterile water. From that biomass, approximately 1 g was transferred into 3 different sets of four Erlenmeyer flasks, each containing 100 ml of the LCM with the following conditions: copper sulphate 1 μM, 5 μM and control without the inducer. The twelve Erlenmeyer flasks were then incubated for a period of 48, 72, 96 and 120 h, at 150 rpm and 28°C. For each condition, total RNA was extracted according Chomczynski and Sacchi (1987).

For the cDNA syntheses were used the SuperScript™ III Reverse Transcriptase kit (Invitrogen). PCR amplifications were performed as previously described in Section 2.3.1 (x = Lcc: 48; β-tubulin = 47). PCR reactions were done in a final volume of 50 μl, containing: 2 μl of cDNA of those samples, 1 μl of 200 mM deoxynucleotide triphosphates (Promega), 3 μl of MgCl_2_ solution (25 mM), 10 μl of GoTaq Flexi buffer (10×), 1 μl of each pair of primers (10 mM), 0.25 μl of enzyme GoTAQ Hot Start Polymerase (Promega) and MilliQ water. PCR products were separated by electrophoresis. Amplified products were visualized by using Gel Doc XR System (Bio-Rad). The quantification of expression levels of the gene was performed by densitometry. Documented images of the amplicons were treated with the support of the program ImageJ 1.44f (http://imagej.net/). Results were normalized by densitometry according to the constitutive gene expression of β-tubulin. Thus, it was possible to establish the ratio between the Lcc gene expressions relative to β-tubulin under different conditions.

### Analytical methods

#### Biomass

For the dry weight biomass measurement the samples were filtered on filter paper n° 41 (45-mm of diameter) under vacuum and kept at 105°C for 8 hours. Biomass was calculated by subtracting the initial weight from the final weight.

### Enzymatic assays

The enzymatic activities of Lcc (ε_525nm_ = 65000 M^−1^ cm^−1^) and proteases (ε_440nm_ = 4600 M^−1^ cm^−1^) were determined as described by Martins et al. (2003). For each enzymatic activity assay, the same reaction mixtures containing boiled supernatant samples were used as control. One unit (U) of enzyme activity was defined as the amount of the enzyme for changing the absorbance by 0.01 per minute. Enzyme activities of all the samples were expressed as U · l^−1^.

### Determination of total proteins

Total proteins were determined by Bradford method using Coomassie Protein Assay Kit (Thermo Scientific Pierce) according to the manufacturer. A standard calibration curve was constructed with three replicates. Total proteins content were presented as μg·ml^−1^.

## Results

### Effect of copper sulphate on laccase synthesis

Results showed the efficiency of these two fungal strains to synthesize Lcc in a LCM supplemented with different copper sulphate concentrations (Table 1). *Nigrospora* sp. CBMAI 1328 displayed the greatest amount of Lcc activity (25.2 U·l^−1^) when the medium was supplemented with copper sulphate at 5 μM. In the presence of the highest copper sulphate concentrations (10 μM) there was a decrease in the Lcc activity, indicating a possible enzymatic inhibition. In contrast, *Arthopyrenia* sp. CBMAI 1330 presented highest (3.9 to 7.7 U·l^−1^) Lcc activities without copper sulphate (the control). The Lcc production by this fungus in the presence of copper sulphate (1–10 μM) ranged from 1.7 to 5.5 U·l^−1^. In fact, Lcc activity decreased with increased copper sulphate (Table 1).

**Table 1:** **Data related to the biomass, total protein and enzymatic activity by the selected marine fungi after incubation in LCM supplemented with different [CuSO**_**4**_ **] during 7 days at 28°C and 150 rpm**

**Fungal strain**	**Time (d)**	Control*			**[CuSO**_**4**_ **] 1 μM**			**[CuSO**_**4**_ **] 5 μM**			**[CuSO**_**4**_ **] 10 μM**		
		Biomass** (g·l ^**−1**^ **)**	**Total protein (μg·ml** ^**−1**^ **)**	**Lcc (U·l** ^**−1**^ **)**	**Biomass (g·l** ^**−1**^ **)**	**Total protein (μg·ml** ^**−1**^ **)**	**Lcc (U·l** ^**−1**^ **)**	**Biomass (g·l** ^**−1**^ **)**	**Total protein (μg·ml** ^**−1**^ **)**	**Lcc (U·l** ^**−1**^ **)**	**Biomass (g·l** ^**−1**^ **)**	**Total protein (μg·ml** ^**−1**^ **)**	**Lcc (U·l** ^**−1**^ **)**
***Nigrospora*** **sp.** CBMAI 1328	**1**	0.3	6.0	1.2	0.1	5.6	2.7	1.4	6.4	5.5	0.1	5.1	1.4
	**3**	1.6	5.2	1.8	2.7	5.8	2.8	3.8	6.5	5.6	3.6	5.2	4.3
	**5**	1.8	5.7	6.5	3.9	5.4	9.5	4.0	7.0	25.2	4.0	5.6	8.5
	**7**	2.6	8.5	5.3	4.2	6.6	6.8	4.5	7.0	22.5	4.5	6.4	7.0
***Arthopyrenia*** **sp** ***.*** CBMAI 1330	**1**	0.6	2.6	3.9	0.7	3.0	4.4	0.5	4.8	1.8	0.7	4.8	1.7
	**3**	1.6	2.6	7.5	0.9	4.6	5.2	0.7	5.4	3.0	0.9	5.0	2.1
	**5**	2.1	3.3	7.7	2.9	5.5	5.1	2.9	5.5	3.3	2.5	5.1	2.8
	**7**	2.0	3.4	6.0	2.8	5.4	4.4	2.3	6.5	3.5	2.2	5.2	2.7

*Control = the same culture conditions without CuSO_4_.

**Biomass = dry matter.

The relationship between copper induction, biomass and total protein was evaluated in the present study (Table 1). *Arthopyrenia* sp. CBMAI 1330 presented no significant variation in the biomass production for the assays with and without CuSO_4_. However, an increasing in total protein was observed for all concentrations of CuSO_4_ in comparison to the control. In contrast, the *Nigrospora* sp. CBMAI 1328 produced more biomass in the presence of CuSO_4_. In the culture conditions where the best results of laccase activities were achieved by this fungus (5 μM of CuSO_4_), the total protein and biomass obtained were highest than that one from the other assays, with the exception of the total protein produced in the control after 7 days and the biomass produced in the presence of CuSO_4_ 10 μM after 5 and 7 days.

### Laccase genes expression

**Figure 1:**
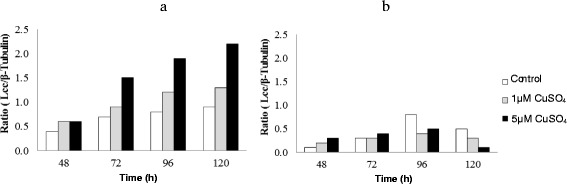
**Detection of Lcc gene expression by** ***Nigrospora*** **sp. CBMAI 1328 (a) and** ***Arthopyrenia*** **sp. CBMAI 1330 (b) in different conditions by using RT-PCR.** The Lcc gene expression was determined by the relative intensity based on the ratio between the Lcc gene expressions relative to β-tubulin.

By using the RT-PCR approach, the levels of Lcc gene expression by the *Nigrospora* sp. CBMAI 1328 and *Arthopyrenia* sp. CBMAI 1330 were determined. Laccase gene expression was detected in the presence and absence of CuSO_4_ (Figure 1). These results allow the inference that these fungi the enzyme is constitutively and inductively expressed. The highest rate of Lcc gene expression by *Nigrospora* sp. CBMAI 1328 was achieved in the presence of 5 μM CuSO_4_ after 120 h of incubation in LCM. In these same conditions this fungus produced the higher level of laccase (25.2 U·L^−1^), as showed in Table 1. For *Arthopyrenia* sp. CBMAI 1330 higher Lcc gene expression was detected in LCM without supplementation of CuSO_4_ (control) after 96 h of incubation (Figure 1b). Coincidently, the higher rates of Lcc activity by this fungus were also observed in submerged LMC without CuSO_4_ after 96 and 120 h of incubation (Table 1).

### Laccase genes characterization

Lcc genes from *Nigrospora* sp. CBMAI 1328 and *Arthopyrenia* sp. CBMAI 1330 were characterized based on cloning and sequencing analysis. An amount of 17 and 20 clone sequences were recovered from the fungi *Nigrospora* and *Arthopyrenia*, respectively. Amongst all of them, only tree clones presented similarity with sequences from fungal Lcc genes (Table 2): (i) two (C9 and F9) from *Arthopyrenia* sp. CBMAI 1330 which showed high similarity (between 70 to 100%) with a Lcc gene from *Clavariopsis aquatica*, a marine ascomycete fungus representative of order *Microascales* and, (ii) one (A1) from *Nigrospora* sp. CBMAI 1328 that showed 45% similarity with the fungus *Stagonospora* sp., a representative of order *Pleosporales* that can be found in marine environments such as swamps (Lyons et al. 2003).

**Table 2:** **Similarity of clones recovered by PCR of laccase gene from strains** ***Nigrospora*** **sp. CBMAI 1328 and** ***Arthopyrenia*** **sp. CBMAI 1330**

**CBMAI**	**Clone**	**Size intron (pb)**	**ID**	**Similarity (%)**
1328	A1	0	*Stagonospora* sp. (AAN17288)	45
1330	C9	47	*Clavariopsis aquatica* (ACR20672)	71
	F9	47	*Clavariopsis aquatica* (ACR20672)	100

**Figure 2:**
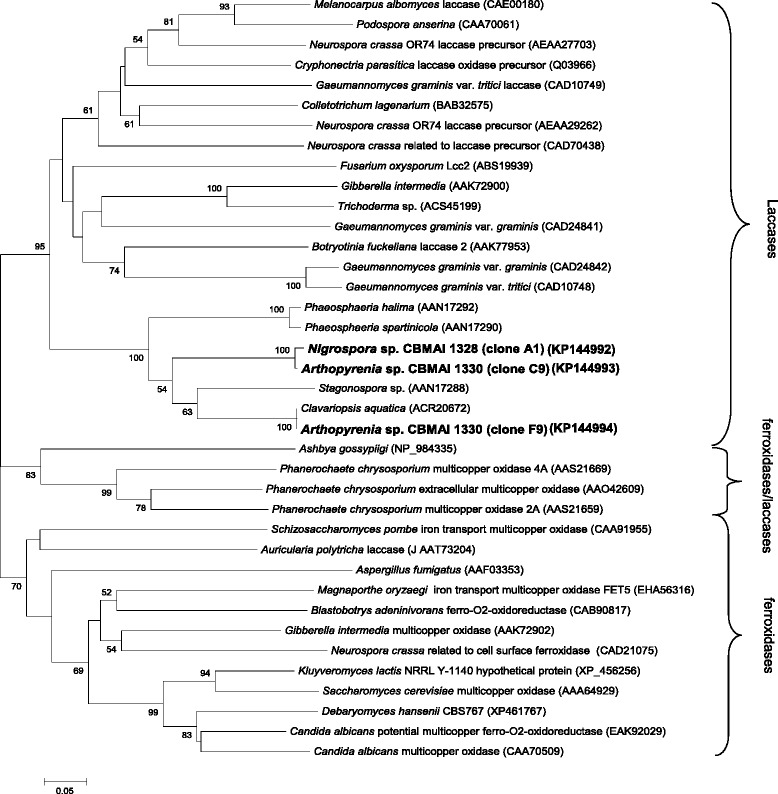
**Phylogenetic tree constructed based on the amino acid alignment of protein sequences of fungal Lcc genes.** Bootstrap with 1000 replicates was performed by analysis of P-distance. Values greater than 50% are indicated at the nodes of the branches. The horizontal bar represents a distance of 0.05 amino acid substitutions per site.

Data derived from phylogenetic analyses showed that only the sequence of clone F9 formed a cluster with the Lcc gene from fungus *Clavariopsis aquatica*. The other two sequences (from clone C9 and A1) clustered together and separated from the sequences recovered from GenBank database (Figure 2).

**Figure 3:**
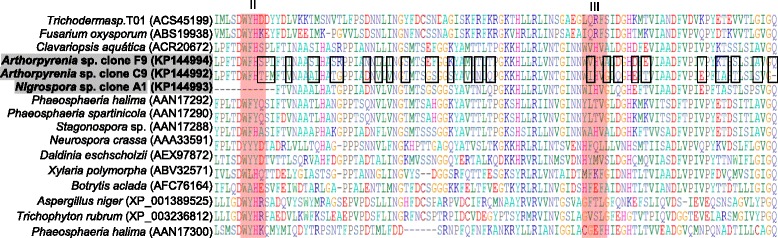
**Lcc gene sequences from** ***Nigrospora*** **sp. CBMAI 1328 (clone A1) and** ***Arthopyrenia*** **sp. CBMAI 1330 (clones F9 and C9) aligned together with sequences recovered from GenBank.** The red boxes show the possible II and III regions of binding of copper ions and the black rectangles show the differences between the amino acids sequences from clones F9 and C9.

**Figure 4:**
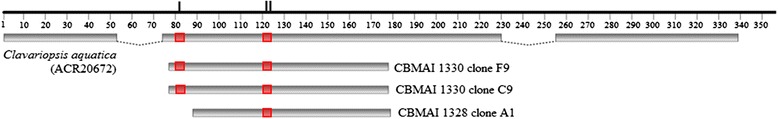
**Comparative schematics of Lcc genes from** ***Nigrospora*** **sp. CBMAI 1328 (clone A1),** ***Arthopyrenia*** **sp. CBMAI 1330 (clones F9 and C9) and** ***Clavariopsis aquatic*** **(ACR20672).** The red boxes show the possible copper binding regions (I and II).

Molecular characterisation of Lcc genes from the *Nigrospora* sp. CBMAI 1328 and *Arthopyrenia* sp. CBMAI 1330 are showed in Figure 3. Comparative schematics of Lcc genes from both marine-derived fungi and *Clavariopsis aquatica* are presented in Figure 4.

## Discussion

Copper is an essential micronutrient for most living organisms, and requirements by fungi are usually satisfied by very low concentrations of the metal, in the order of 1–10 μM. Copper present in higher concentrations of its free, cupric form is extremely toxic to fungal cells (Galhaup and Haltrich 2001). The mechanism of the Lcc induction by copper is associated with its role in the Lcc active centre, and its participation in the regulation of Lcc genes transcription and post-transcription modifications; whereas, copper toxicity is attributed to the interaction of copper ions with proteins, enzymes, nucleic acids, and metabolites associated with cell functions and viability, and due to oxidative (Kannaiyan et al. 2012).

Copper is also often a strong inducer of Lcc gene transcription, and this may be related to a defence mechanism against oxidative stress caused by free copper ions (Viswanath et al., 2008). In a recent study, Pezzella et al. (2013) performed a transcriptional analysis of nine *Pleurotus ostreatus* Lcc genes by RT-PCR in different growth conditions. The authors concluded that the addition of copper to the culture medium resulted in strong induction of *lcc9/lcc10* and *lcc2* genes, and a lesser induction of other *lcc* genes. In some cases these responses were dependent on the time of growth. Santo et al. (2012) evaluated the degradation of polyethylene by polyethylene-degrading *Rhodococcus ruber* and concluded that copper markedly affected the induction and activity of Lcc, resulting in polyethylene degradation. The mRNA quantification by RT-PCR revealed a 13-fold increasing in Lcc mRNA levels from copper-treated cultures in comparison with the untreated control. Additionally, the authors emphasise that the addition of copper to *R. ruber* cultures containing polyethylene, enhanced by 75% the biodegradation of this compound.

According to Kannaiyan et al. (2012), the induction of *Dichomitus squalens* Lcc activity required copper sulphate addition as low as 0.06 mM. Palanisami and Lakshmanan (2011) using the marine filamentous non-heterocystous cyanobacterium *Phormidium valderianum* observed a negative effect of Lcc activity when the concentration of copper was greater than 10 μM and, consequently, a gradual decrease in the rate of Poly-R478 dye decolourisation. Singhal et al. (2009) reported a seven fold enhancement of Lcc production by *Cryptococcus albidus* after the optimizing of the growth media, which contained 2 mmol·l^−1^ of copper sulphate. However, for *C. albidus,* Lcc production was inhibited with increasing copper sulphate concentration. Cordi et al. (2007) showed that the addition of 0.07-0.1 mM copper sulphate during the cultivation of *Trametes versicolor* promoted higher Lcc activity, reaching a maximum value of 40.44 U·l^−1^ on day 12 of incubation. In contrast, in the absence of this inductor the values detected for Lcc were considered insignificant. In Sun et al. (2009) the relationship between CuSO_4_ and fungal biomass was discussed. The authors observed that at high levels of copper sulphate (0.8 mM) the activity of crude Lcc was significantly inhibited possibly due to the weak fungal growth.

Data derived from phylogenetic analyses may be explained by the absence of sequences close to the *Nigrospora* sp. CBMAI 1328 and *Arthopyrenia* sp. CBMAI 1330 Lcc genes or may illustrate the recovery of potential new Lcc genes from fungi associated with marine sponges. In a previous work carried out by our research group, three putative new Lcc genes were detected in a marine-derived basidiomycete (Bonugli-Santos et al. 2010). In addition, Lyons et al. (2003) identified 15 distinct sequences of Lcc from ascomycetes isolated from salt marshes in the southeastern U.S.

A study by Hoegger et al. (2006) reported that the composition of multicopper oxidases in fungal species can be very different and some species appear to encode only one type of enzyme such as, ferroxidases, as well as other species can produce other types of enzymes belonging to this family. Based on this report, representative sequences of Lcc, ferroxidase and “ferroxidases/Lcc” (enzymes with strong ferroxidase activity and weak Lcc activity) were recovered and aligned with *Nigrospora* sp. CBMAI 1328 and *Arthopyrenia* sp. CBMAI 1330 Lcc protein sequences (Figure 2). Results from phylogenetic analyses showed that Lcc genes from the studied marine fungi formed a cluster with protein sequences that encoding strictly Lcc enzymes. In addition, all Lcc sequences from *Nigrospora* sp. CBMAI 1328 and *Arthorpyrenia* sp. CBMAI 1330 grouped with Lcc protein sequences from ascomycetes derived from marine environments.

Since the primer set used was designed to be specific for fungal Lcc and targeted conserved sequences around two pairs of histidine involved in two of the four copper binding regions designated II and III, the alignment of protein sequences from *Arthopyrenia* clones F9 and C9 and from *Nigrospora* clone A1 may be related to regions II and III of binding copper ions (Figures 3 and 4).

The comparison between *Arthopyrenia* clones F9 and C9 protein sequences (Figure 3) and gene sequences alignment (data not shown) revealed that, five different nucleotides and 29 different amino acids were found despite these two Lcc genes being very similar. These results confirm the hypothesis of divergent copies of Lcc genes within the genome of this fungus, where more than one isoform can be expressed under specific experimental conditions.

The presence of multiple Lcc genes in the fungal genome has been discussed previously. According to Ramos et al. (2011), this is probably due to the different physiological roles played by their products and their regulation by environmental conditions. Susla et al. (2007) reported that the diversity of genes can be attributed to post-transcriptional modifications of enzymes within the cell. In conclusion, the use of Lcc gene libraries from filamentous fungi has been a powerful technology for the characterization of this gene (D’Souza et al. 1996; Dedeyan et al. 2000; Dubé et al. 2008).

The detection of two different Lcc genes in the genome of *Arthopyrenia* sp. CBMAI 1330, suggests the presence of two isoforms. For this fungus, the expression of Lcc was higher when the copper ion was absent in the culture medium. However, in the screening experiments (data not shown) Lcc activity in the range of 15 U·l^−1^ was achieved when 1 mM of CuSO_4_ was added to de medium. It is reasonable to speculate that a different isoform may have been expressed by the use of copper ion.

The present work revealed how Lcc activity from two marine-derived fungal strains, identified as belonging to the genera *Nigrospora* and *Arthopyrenia* could be increased by the addition of copper sulphate. Molecular and phylogenetic analyses allow the classification of these enzymes and suggest the presence of putative new enzymes. In addition, Lcc from the *Nigrospora* sp. CBMAI 1328 and *Arthopyrenia* sp. CBMAI 1330 clustered with other Lcc from fungi that inhabit marine environments.

Fungi from marine environments can produce different enzymes from those produced by the same terrestrial species since they are adapted to oceans where in some cases includes extremes of pH and salinity. The two marine-derived fungal strains studied could be considered strategic for the biotechnology, since they showed to be potential genetic resources, which could be applied in saline environments and/or technological processes.
